# Exploration of post-PEG precipitation TSH recovery in hypothyroid patients

**DOI:** 10.3389/fendo.2025.1715348

**Published:** 2025-11-07

**Authors:** Jing Yin, Zhanjun Mei, Bo Zhang, Fang Tang

**Affiliations:** Department of Nuclear Medicine, The Second Affiliated Hospital of Chengdu Medical College Nuclear Industry 416 Hospital, Chengdu, Sichuan, China

**Keywords:** thyroid stimulating hormone, polyethylene glyco, recovery rate, hypothyroidism, subclinical hypothyroidism

## Abstract

**Introduction:**

Thyroid disorders are among the most common endocrine diseases, and their diagnosis and monitoring rely heavily on laboratory testing. However, immunoassays used to assess thyroid function are susceptible to various types of interference, which can affect clinical decision-making. This study aimed to establish a novel method for evaluating the potential interference of serum macromolecules in the detection of Thyroid Stimulating Hormone (TSH).

**Materials and methods:**

A total of 160 patients (87 with hypothyroidism and 73 with subclinical hypothyroidism) from the Nuclear Industry 416 Hospital between July 1, 2023 and November 30, 2023, were enrolled as the experimental group. Additionally, 160 healthy individuals were randomly selected from the health examination center as the control group. Samples were treated using polyethylene glycol (PEG) precipitation method, and TSH recovery rates were calculated.

**Results:**

Significant differences in TSH recovery rates were observed among the hypothyroidism group (35.0% ± 13.3%), subclinical hypothyroidism group (30.1% ± 7.9%), and control group (56.9% ± 12.4%) (P< 0.05). A TSH recovery rate cutoff-value of 28% was established. The incidence of macromolecular interference was 36.6% in the hypothyroidism group and 39.7% in the subclinical hypothyroidism group, with no significant difference between the two groups (P = 0.771). All control group participants had TSH recovery rates >28%. In hypothyroid samples with TSH recovery rates<28%, a positive correlation was found between TSH recovery and FT3 levels (P = 0.027, Pearson correlation coefficient = 0.396).

**Conclusions:**

This study provides a new reference for the clinical evaluation of TSH. When TSH recovery rates are below 28% in patients with hypothyroidism or subclinical hypothyroidism, the presence of serum macromolecules should be considered.

## 1 Introduction

Thyroid function testing is a crucial tool for evaluating thyroid disorders. Among its components, Thyroid Stimulating Hormone (TSH) serves as a central indicator for diagnosis and monitoring due to its high sensitivity and specificity (1, 2). TSH concentrations in the blood exhibit a logarithmic relationship with thyroid hormone levels, meaning even minor fluctuations in hormone levels can trigger significant changes in TSH. Elevated TSH is the most common indicator of hypothyroidism, while decreased Free Triiodothyronine (FT3) and Free Thyroxine (FT4) levels can further confirm the diagnosis (3, 4). In patients with subclinical hypothyroidism, the hormonal profile is characterized by elevated TSH levels with FT3 and FT4 remaining within the normal range (5). Additionally, testing for anti-thyroglobulin antibody (TgAb) and anti-thyroid peroxidase antibody (TPOAb) aids in the diagnosis.

Immunoassay techniques, including direct chemiluminescence immunoassay, enzyme chemiluminescence immunoassay, and electrochemiluminescence immunoassay, have become the preferred method for measuring thyroid hormone levels due to their high sensitivity and specificity (6). However, in clinical testing, TSH levels are often subject to interference from macromolecular substances such as macro-TSH (m-TSH), human anti-mouse antibodies (HAMA), heterophilic antibodies, autoantibodies, anti-ruthenium antibodies, and rheumatoid factor (7–14). Among these, m-TSH is a high-molecular-weight polymer composed of monomeric TSH and its autoantibodies (15–17). Although m-TSH lacks biological activity (18), it can be detected by the widely used chemiluminescent immunoassays (CLIAs). The system mistakenly identifies it as biologically active monomeric TSH, resulting in falsely elevated TSH measurements (19). Furthermore, monomeric TSH (approximately 30 kDa) is easily filtered and excreted by the kidneys, while macromolecules like m-TSH (greater than 150 kDa) accumulate in the peripheral circulation due to impaired filtration. This interference can persistently affect clinicians’ diagnosis and potentially lead to unnecessary treatments, such as increased Levothyroxine (L-T4) intake causing exogenous hyperthyroxinemia (20). Levothyroxine (L-T4) is the gold standard for treating hypothyroidism (21–24). According to a joint consensus statement from the American Association of Clinical Endocrinologists and the American Thyroid Association, after initiating L-T4 therapy, dose adjustments should be guided by TSH levels. The initial dose increment is typically 12.5 – 25 µg per day until the target TSH range is achieved. If TSH remains persistently elevated despite high-dose L-T4 treatment, optimizing the administration method or further increasing the dose should be considered (25). Notably, m-TSH exhibits laboratory features similar to those of subclinical hypothyroidism (26). Currently, there are no commercial TSH testing platforms available that avoid cross-reaction with m-TSH (27), and m-TSH may persist in patients long-term, continuously interfering with test results.

The polyethylene glycol (PEG) precipitation method is widely employed in prolactin (PRL) testing to mitigate interference from macro-PRL (m-PRL) (28, 29). Given the structural similarity between m-TSH and m-PRL, this method has been adapted for TSH testing in international studies (30–34). However, there has been a lack of clinical studies using PEG precipitation to assess macromolecular interference in China (30). Due to heterogeneity in TSH levels among different populations (35), the direct application of internationally recommended TSH recovery cut-off values poses challenges for clinical practice. This study aims to propose a region-specific cut-off value for TSH recovery rate and to evaluate the clinical utility of TSH recovery analysis in cases where hypothyroid or subclinical hypothyroid patients exhibit persistently elevated TSH levels and require higher-than-expected LT4 doses to achieve therapeutic targets.

## 2 Materials and methods

### 2.1 Subjects

A total of 160 patients who visited Nuclear Industry 416 Hospital between July 1, 2023 and November 30, 2023, were enrolled in the experimental group, including 87 patients with hypothyroidism and 73 with subclinical hypothyroidism. Meanwhile, data from 445 healthy individuals were collected from the hospital’s health management center. Stratified random sampling was applied to select 160 subjects as the control group. According to the Chinese Guidelines for the Diagnosis and Management of Thyroid Diseases (36), the reference range for TSH in the general population is 0.3-4.5 mIU/L. The guidelines emphasize that each laboratory should establish its own reference interval for TSH. The reference intervals established in our laboratory are as follows:

Ages 2-12: 0.64-6.27 mIU/L.

Ages 12-18: 0.51-4.94 mIU/L.

Ages 18 and above: 0.55-4.78 mIU/L.

The inclusion criteria for the experimental group were:

(1) Age 18 years or older.

(2) Clinical diagnosis of hypothyroidism, characterized by elevated TSH levels above the upper limit of the reference interval (> 4.78 mIU/L), accompanied by free triiodothyronine (FT3) and free thyroxine (FT4) levels below the lower limit of the reference interval; or diagnosis of subclinical hypothyroidism, defined as elevated TSH levels above the upper reference limit (> 4.78 mIU/L) with normal FT3 and FT4 levels (37, 38).

(3) Receiving levothyroxine (LT4) treatment.

The control group consisted of healthy individuals with no history of thyroid disease, no previous treatment for thyroid disorders, and hormone levels (including TSH) within the reference intervals (39). Individuals with repeated tests, visibly lipemic, icteric, or hemolyzed samples, or those with incomplete baseline information were excluded. The following data were collected from included subjects: gender, age, and thyroid-related indicators, including TSH, FT3, FT4, total triiodothyronine (TT3), total thyroxine (TT4), TgAb, and TPOAb levels.

### 2.2 Methods

All blood samples were collected in the morning (between 8:00 AM and 11:00 AM) after an overnight fast, 5 mL of venous blood was collected from the subjects. Venous blood samples were collected using the MicralD MH-L 700 vacuum blood collection system (Chongqing Weibiao Technology Co., Ltd., Chongqing, China), with BD Vacutainer serum separation tubes (Becton, Dickinson and Company, Franklin Lakes, NJ, USA) and matching disposable retractable needle-stick prevention venous blood collection devices (Shanghai Baoshun Medical Devices Co., Shanghai, China). The sample was inverted and mixed thoroughly, then allowed to stand for 20 min. It was subsequently centrifuged at 2, 000 × *g* for 10 min, and the separated serum was stored at -20°C for further testing. The levels of hormones such as TSH were measured using the Siemens Atellica^®^IM1600 analyzer (Siemens Healthineers, Erlangen, Germany) and corresponding matched test kits (direct chemiluminescence method). TgAb and TPOAb levels were determined using the Elecsys Systems Modular Analytics e801 (Roche Diagnostics, Basel, Switzerland) and Roche-matched reagent kits. After testing, all subject samples were further processed using the PEG precipitation method to assess the presence of macromolecules such as m-TSH and heterophilic antibodies. A 25% aqueous solution of PEG6000 was prepared by dissolving 2.5 g of PEG6000 (Tianjin Zhiyuan Chemical Reagent Co, Tianjin, China) in 10 mL of deionized water. Then, 200 µL of serum sample was mixed with an equal volume (200 µL) of either 25% PEG6000 solution (TSH·PEG) or deionized water (TSH·H_2_O). After thorough mixing, the samples were centrifuged at 2, 000 × *g* for 5 min, and the supernatant was collected to determine the concentrations of TSH·PEG and TSH·H_2_O. The recovery rate of TSH was calculated using Equation 1 (40). Calibration was performed strictly per manufacturer protocols using specific calibrators: TSH calibrators (Siemens, USA, lots CH11 & CH19) and Multi-Assay Calibrator A for TT3, TT4, FT3, and FT4 assays (Siemens, USA, lot number was part of routine records but not specifically tracked for this retrospective analysis). All calibrations were performed under our standard operating procedures which mandate the use of valid, manufacturer-provided calibrators. Quality control was maintained using Immunoassay Universal Control (Bio-Rad, USA, lot 40410), which demonstrated excellent precision with an intra-assay CV of 4.5% and an inter-assay CV of 5.1%. All pre-test quality control results were within acceptable ranges, confirming the reliability of the data throughout the study.

(1) $$\text{Recovery rate}=\frac{\text{TSH}\cdot \text{PEG}}{\text{TSH}\cdot {\text{H}}_{2}\text{O}}\times 100\%$$

### 2.3 Statistical analysis

Statistical analyses were performed using Version 26.0 of IBM SPSS Statistics for Windows (IBM Corp., Armonk, NY, USA) and figures were generated using Origin 2018 64bit (OriginLab Corp., Northampton, MA, USA). The normality of continuous data was assessed using the Kolmogorov-Smirnov (K-S) test. Data that conformed to a normal distribution are presented as mean ± standard deviation (Mean ± SD), while Pearson correlation analysis was used to examine data correlations. Multiple linear regression analysis was further employed to assess the independent effects of sex and age (included as covariates) on the TSH recovery rate. Continuous variables (age, TT3, TT4, FT3, FT4) were compared using independent t-tests; TSH levels and TSH recovery rates were analyzed by ANOVA; and categorical data (sex and macromolecular incidence) were assessed using χ^2^ tests, with significance threshold set at α = 0.05 (two-sided test; P< 0.05 deemed statistically significant).

The research related to human use has complied with all the relevant national regulations, institutional policies, and in accordance with the tenets of the Helsinki Declaration, and has been approved by the authors’ Institutional Review Board or equivalent committee (the Medical Ethics Committee of Nuclear Industry 416 Hospital, YJ-2024-075-01).

## 3 Results

### 3.1 General clinical characteristics

The general clinical characteristics of the subjects and their levels of TSH, TT3, TT4, FT3, FT4, TgAb, and TPOAb are detailed in **Table 1**. In the experimental group, hypothyroid patients accounted for 54.4% (87/160), among whom 34.5% were male; subhypothyroid patients constituted 45.6% (73/160), with 25.7% being male. In the control group composed of healthy individuals, males accounted for 25.0%. Statistical analysis showed no significant differences between the experimental and control groups in terms of gender (P = 0.381) or age (P = 0.283, F = 4.509, t = 1.075, 95%[CI]: -1.141 - 3.891) distributions. Multiple linear regression analysis clearly demonstrates that, after accounting for the potential confounding effects of age and sex, the core variable “Group” remains a robust independent predictor of the TSH recovery rate (Beta = 13.27, P< 0.001). Significant differences (P< 0.05) were observed in the levels of TSH, TT3, TT4, FT3, and FT4 among the groups.

**Table 1:** General clinical characteristics and hormone levels in the experimental and control groups.

Parameter	Experimental group		Control group	P value
	Hypothyroid group	Subhypothyroid group		
Sample N	87	73	160	–
Sex (male/female)	30/57	18/55	40/120	0.381^a^
	48/112			
Age	40.2 ± 12.7	38.3 ± 11.6	37.9 ± 10.6	0.283^b^
	39.3 ± 12.2			
TSH (mIU/L)	46.96 ± 36.72	12.92 ± 9.07	2.38 ± 0.89	<0.001^c^
TT3 (nmol/L)	1.04 ± 0.96	1.39 ± 0.23	–	0.004^d^
TT4 (nmol/L)	48.22 ± 26.62	98.59 ± 16.75	–	<0.001^d^
FT3 (pmol/L)	3.04 ± 1.09	4.26 ± 0.44	–	<0.001^d^
FT4 (pmol/L)	8.58 ± 3.76	15.24 ± 1.94	–	<0.001^d^
TgAb	–	–	17.05 ± 11.28	–
TPOAb	–	–	11.83 ± 3.57	–

a: A χ^2^ corrected test was performed after weighting the gender frequencies of the experimental and control groups, with a two-sided significance level of P > 0.05 indicating that there is no significant difference in gender between the two groups.

b: A t-test was performed on the age of the experimental and control groups at the 95% confidence interval, with P > 0.05 not being a significant difference.

c: One-way ANOVA indicated significant differences in TSH levels among the hypothyroid, subhypothyroid, and control groups.

d: Independent t-tests demonstrated statistically significant differences in TT3, TT4, FT3, and FT4 levels between the hypothyroid and subhypothyroid groups (P< 0.05).

### 3.2 Analysis of TSH recovery rate

**Figure 1:**
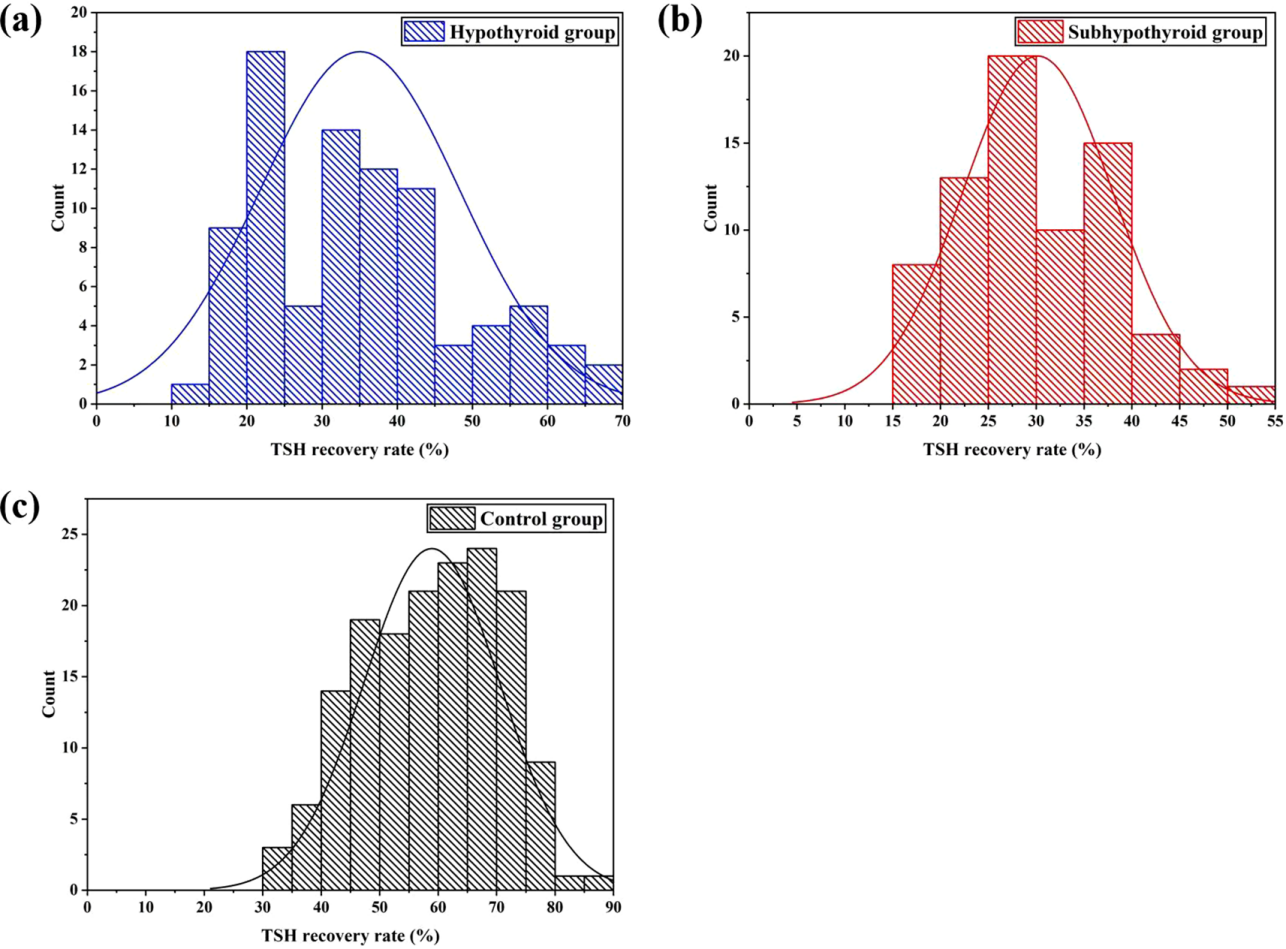
Distribution of TSH recovery rate after PEG precipitation [**(a)** hypothyroid group; **(b)** subhypothyroid group; **(c)** control group].

**Figure 1** illustrates the distribution of TSH recovery rates across the groups. K-S normality tests were performed at the significance level of P = 0.05. The results indicated that the data in all groups were normally distributed: the hypothyroid group (P = 0.223, 95%[CI]: 32.2% - 37.9%), the subhypothyroid group (P = 0.871, 95%[CI]: 28.3% - 32.0%), and the control group (P = 0.347, 95%[CI]: 57.1% - 60.7%). The subhypothyroid group exhibited the lowest lower limit of the 95% confidence interval, at 28.3%. Based on these findings, a cut-off value of 28% was established for the TSH recovery rate in this study. When the TSH recovery rate falls below this threshold, it suggests potential interference in the TSH assay due to macromolecular substances such as m-TSH or heterophilic antibodies.

As shown in **Table 2**, the TSH recovery rate was 35.0% ± 13.3% in the hypothyroid group, 30.1% ± 7.9% in the subhypothyroid group, and 58.9% ± 12.4% in the control group. The TSH recovery rate was significantly lower in the experimental groups than in the control group (P< 0.001). Further subgroup analysis within the experimental groups revealed that the subhypothyroid group had a significantly lower TSH recovery rate compared to the hypothyroid group (P = 0.010). Among samples with a TSH recovery rate below 28%, t-test results indicated no significant difference in TSH recovery between hypothyroid patients (21.5% ± 3.1%) and subhypothyroid patients (22.6% ± 3.4%) (P = 0.469). Similarly, no significant difference was observed in the macromolecular incidence between the hypothyroid group (36.6%) and the subhypothyroid group (39.7%) (P = 0.771, 95%[CI]: -2.735 - 0.614).

**Table 2:** Data analysis of subjects’ TSH recovery rate and macromolecular incidence.

Parameter	Experimental group		Control group	P value
	Hypothyroid group	Subhypothyroid group		
Recovery rate (%)	35.0% ± 13.3%	30.1% ± 7.9%	–	0.010^a^
	35.0% ± 13.3%	30.1% ± 7.9%	56.9% ± 12.3%	<0.001^a^
Samples with a recovery rate<28%				
Sample N	31	29	0	–
Recovery rate (%)	21.5% ± 3.1%	22.6% ± 3.4%	–	0.469^b^
Macromolecular incidence rate (%)	36.6%	39.7%	–	0.771^c^

a: One-way ANOVA of the recovery rates of the hypothyroidism, subhypothyroidism and control groups showed P = 0.010<0.05 for the hypothyroidism and subhypothyroidism groups (95% [CI]: -2.735 - 0.614); P<0.001 for the hypothyroidism and healthy groups (95% [CI]: -24.923 - -18.751), and P = 0.010<0.05 for the subhypothyroidism and healthy groups (95% [CI]: - 29.983 - -23.439) were all P<0.001, so there was a significant difference in the recovery rates of the hypothyroidism group, subhypothyroidism group and control group.

b: In the samples with recovery rate<28%, t-test was performed at 95% confidence interval for the recovery rate of hypothyroidism group and subhypothyroidism group, and P >0.05 was not significant difference.

c: A χ^2^ correction test was performed on the macromolecular incidence in the experimental and control groups, and the two-sided significance of P >0.05 indicated that the incidence of macromolecules in the two groups was not significantly different.

### 3.3 Correlation analysis between hormone levels and TSH recovery rate

**Figure 2:**
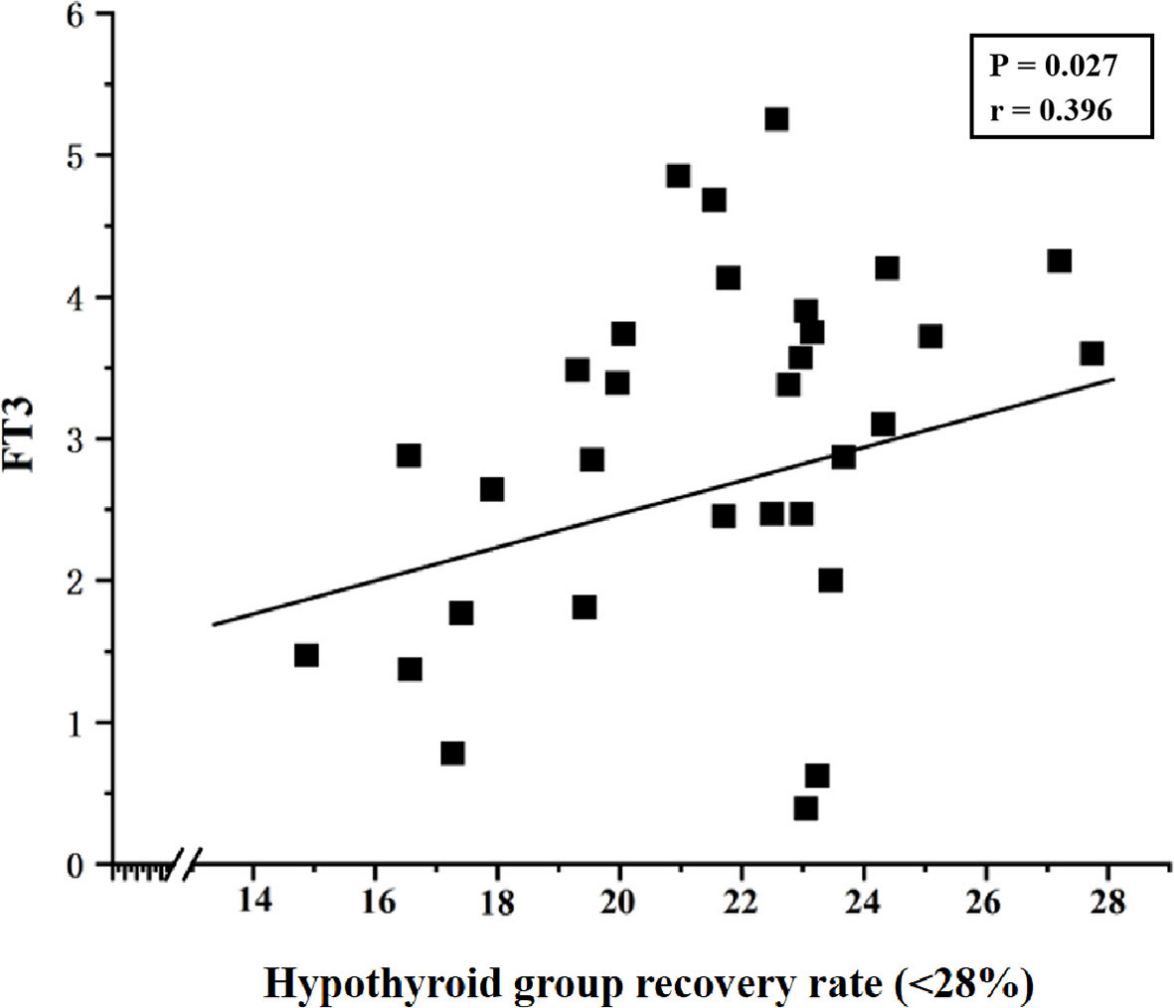
Correlation between TSH recovery rate (<28%) and FT3 levels in the hypothyroid group (P = 0.027, Pearson correlation coefficient (r) = 0.396).

Pearson correlation analysis was performed to assess the association between thyroid function parameters and the TSH recovery rate in the experimental group. Data detailing the correlation between hormone levels (before PEG precipitation) and the TSH recovery rate in the experimental group are presented in **Table 3**. The results indicated no significant correlation between TSH, TT3, TT4, FT3, or FT4 levels and the TSH recovery rate in the experimental group (P >0.05). Further analysis of samples with a TSH recovery rate below 28% revealed a significant correlation between the low recovery rate and FT3 concentration in the hypothyroid group (P = 0.027, Pearson correlation coefficient (r) = 0.396), as shown in **Figure 2**.

**Table 3:** Pearson correlation analysis between hormone levels and TSH recovery rate in the experimental group.

Hormone levels	P value^a^		P value^b^	
	Hypothyroid group recovery rate	Subhypothyroid group recovery rate	Hypothyroid group recovery rate (<28%)	Subhypothyroid group recovery rate (<28%)
TSH (mIU/L)	0.061	0.112	0.848	0.606
TT3 (nmol/L)	0.797	0.679	0.986	0.952
TT4 (nmol/L)	0.473	0.566	0.057	0.412
FT3 (pmol/L)	0.724	0.597	0.027	0.210
FT4 (pmol/L)	0.525	0.176	0.078	0.489

a: Pearson correlation analysis was performed between TSH recovery rates and levels of TSH, TT3, TT4, FT3, and FT4 in the hypothyroid and subhypothyroid groups, respectively. A two-sided significance level of P >0.05 indicated no significant correlation.

b: Further Pearson correlation analysis was conducted between TSH recovery rates<28% and levels of TSH, TT3, TT4, FT3, and FT4 in the hypothyroid and subhypothyroid groups, respectively. A significant correlation was found between TSH recovery rate (<28%) and FT3 level in the hypothyroid group (P<0.05).

## 4 Discussion

This study yielded three principal findings regarding the utility of PEG-precipitated TSH recovery rate in detecting macromolecular interference. Firstly, we established a region-specific cut-off value of 28% for the TSH recovery rate, derived from the distribution in our subhypothyroid cohort. Secondly, the recovery rates were significantly lower in both the hypothyroid (35.0% ± 13.3%) and subhypothyroid (30.1% ± 7.9%) groups compared to the healthy controls (56.8% ± 12.4%), suggesting a high prevalence of potential macromolecular interference in patient populations. Thirdly, application of the 28% cut-off value retrospectively identified illustrative cases where a mismatch between LT4 dosage and TSH levels had been documented. In these cases, subsequent clinical management that happened to incorporate PEG-precipitated TSH results coincided with the normalization of thyroid function in follow-up examinations, illustrating how this metric could prevent excessive LT4 treatment. This provides preliminary evidence that the PEG-precipitated TSH recovery rate can serve as a practical screening tool to circumvent assay interference and guide more precise therapy.

PEG precipitation is widely used as a screening tool for macromolecular substances due to its simplicity and low cost (12). The principle of this method is to precipitate macromolecular complexes and calculate the recovery rate of free TSH after precipitation. A lower recovery rate indicates a higher proportion of macromolecular complexes precipitated by PEG in the sample. For patients with hypothyroidism or subclinical hypothyroidism, the TSH recovery rate after PEG precipitation can serve as an indicator to assess whether TSH levels are affected by macromolecular interference, particularly in cases where TSH levels are elevated but response to thyroid hormone replacement therapy is unsatisfactory. However, Chinese expert consensus has not yet established a specific cut-off value for TSH recovery rate following PEG precipitation (41). The results of this study showed that the TSH recovery rates were 35.0% ± 13.3% in the hypothyroid group, 30.1% ± 7.9% in the subhypothyroid group, and 56.8% ± 12.4% in the healthy control group. The significantly lower TSH recovery rates in hypothyroid and subhypothyroid patients compared to healthy controls may be attributed to immune system dysregulation often seen in these patients (especially in autoimmune thyroid diseases such as Hashimoto’s thyroiditis), which is characterized by elevated autoantibodies like TgAb and TPOAb, and possibly accompanied by the production of macromolecules such as anti-TSH autoantibodies (42). These anti-TSH antibodies can also bind to endogenous TSH to form m-TSH. Furthermore, persistently elevated TSH levels may stimulate the immune system to produce more anti-TSH antibodies through antigen-driven immune responses (43). In contrast, healthy individuals have a stable immune system with minimal anti-TSH autoantibodies; TSH primarily exists in a free monomeric form that is less likely to be precipitated by PEG, resulting in a higher recovery rate.

Subgroup analysis within the experimental group revealed that the subhypothyroid group had a significantly lower TSH recovery rate than the hypothyroid group (P = 0.010). Currently, there is no universally established reference range for TSH recovery rate using PEG precipitation. Mills F et al. (40) conducted a study on 495 samples with TSH concentrations >10 mIU/L and found that TSH recovery rates followed a normal distribution after treatment with 25% PEG solution, with a mean recovery rate of 47.0% ± 11.2% and a 95% reference range (mean ± 2SD) of 25.6% – 69.4%. Based on this, the authors suggested that further investigation for m-TSH is warranted when the TSH recovery rate is<25%. In the present study, however, the lower limit of the 95% confidence interval for the subhypothyroid group (28%) was set as the cut-off value based on the distribution of TSH recovery rates after K-S testing. The results showed that no sample in the control group had a TSH recovery rate below 28%. The incidence of macromolecular interference was 36.6% in the hypothyroid group and 39.7% in the subhypothyroid group, indicating a relatively high likelihood of macromolecular presence in serum. Analyzing TSH recovery rates in hypothyroid and subclinical hypothyroid patients thus holds significant clinical value. Although m-TSH is a rare phenomenon, its long-term presence should not be overlooked. Other factors such as heterophilic antibodies, autoantibodies, and anti-ruthenium antibodies can also interfere with assay results and misguide clinical decisions (7, 13). It is worth noting that besides the TSH recovery rate, the precipitation index has also been used in some studies to detect m-TSH in serum (10). Giusti M (44) found a negative correlation between the PEG precipitation index and FT4 levels in thyroid cancer patients. In this study, correlation analysis indicated no statistically significant relationship between TSH recovery rate and levels of TSH, TT3, TT4, FT3, or FT4 in either hypothyroid or subhypothyroid patients (P > 0.05). However, in samples with TSH recovery rates below 28%, further analysis revealed a positive correlation between TSH recovery rate and FT3 in hypothyroid patients (P = 0.027, Pearson correlation coefficient = 0.396). Although a statistically significant correlation was observed between FT3 and TSH recovery, the strength of this association is weak, and its clinical significance is likely limited. The biological basis for this weak association warrants further investigation.

Currently, domestic research on the use of PEG precipitation to exclude macromolecular interference in TSH testing remains relatively scarce, and both its detection technology and clinical value require further validation. This study established 28% as the cut-off value for TSH recovery rate and applied it in clinical practice. The results revealed that some patients (2 cases) with a TSH recovery rate below 28% exhibited a mismatch between their LT4 dosage and the expected TSH level. A review of these two patients’ medical records over the past two years showed that their TSH levels remained consistently elevated (15–50 mIU/L). Regardless of whether the LT4 dose was increased or decreased, TSH remained high; moreover, increasing the dose led to thyroid hormone levels exceeding the normal range. Their subsequent TSH tests were processed using PEG precipitation, and medication was adjusted based on the post-precipitation TSH level. The results from two follow-up examinations within the last six months showed that both the PEG-precipitated TSH levels (4–5 mIU/L) and thyroid hormone levels were within the normal range. This approach effectively avoided excessive LT4 intake while maintaining the original dosage. Therefore, the analysis of TSH recovery rate provides a valuable supplementary tool to conventional TSH testing in clinical practice.

Of course, this study also has some limitations. Firstly, as a single-center, cross-sectional study, it inherently lacks multi-center validation and longitudinal follow-up. The proposed 28% cut-off for TSH recovery requires external validation in independent cohorts and across different immunoassay platforms. Future work will include long-term, multi-center collaborative studies to address this, employing receiver operating characteristic (ROC) curve analysis with a gold standard method such as gel filtration chromatography to establish a more robust and clinically actionable cut-off value. Secondly, the precise composition of the interfering macromolecular substances remains uncharacterized. Future research should utilize methods like protein A/G precipitation and gel chromatography to identify their specific components and elucidate the underlying immunological mechanisms. Thirdly, potential confounding factors, such as the dosage and duration of LT4, which may influence serum TSH levels and immune responses, were not systematically controlled. Prospective studies designed to rigorously collect and analyze these treatment variables are warranted.

## 5 Conclusions

In clinical practice, it is difficult to determine whether thyroid function testing is affected by macromolecular interference based solely on hormone levels such as TSH, T3, T4, FT3, and FT4. Therefore, for general health screening populations and hypothyroid/subclinical hypothyroid patients whose medication response aligns with expected hormone levels, conventional thyroid function testing procedures are sufficient. For hypothyroid/subclinical hypothyroid patients receiving LT4 therapy whose dosage does not match the expected hormone levels, the PEG-precipitated TSH recovery rate serves as a useful screening tool for identifying potential macromolecular interference. However, its direct application for guiding LT4 dosage adjustments requires validation through prospective, patient outcome-oriented studies.

## Data Availability

The original contributions presented in the study are included in the article/supplementary material, further inquiries can be directed to the corresponding author/s.
